# Prednisolone and enoxaparin (clexane) therapy (‘the Bondi protocol’) for repeated IVF failure

**DOI:** 10.1111/aji.13616

**Published:** 2022-09-11

**Authors:** Gavin Sacks, Jessica Zhang

**Affiliations:** ^1^ IVFAustralia Sydney Australia; ^2^ Department of Women’s and Children’s Health University of New South Wales Sydney Australia; ^3^ St George Hospital and Royal Hospital for Women Sydney Australia

**Keywords:** Bondi protocol, immune therapy, natural killer cells, repeated IVF failure

## Abstract

**Problem:**

What is the impact of an empirical immune therapy protocol of prednisolone and enoxaparin (clexane) (the ‘Bondi protocol’) on women with repeated in vitro fertilization (IVF) failure?

**Method of study:**

This was a retrospective review of live birth outcomes conducted on all transfer cycles performed by a single clinician (GS) at IVFAustralia between February 2016 and April 2020. This study consisted of 1786 transfer cycles, including 460 cycles treated with the Bondi protocol and 1326 without. Women with repeated IVF failure were given the Bondi protocol based on blood NK cell activity. Primary outcome was live birth and statistical analysis was performed with GraphPad Prism software with significance for *P*‐values < .05.

**Results:**

Overall ‘Bondi’ and ‘normal’ protocol cycles had similar rates of IVF/ICSI, fresh/frozen transfers and use of preimplantation genetic testing (PGT). Women given the Bondi protocol were older, had more previous cycles and had higher blood NK cell activity. There was no significant difference in live birth rates (Bondi 26%, normal 28%). Bondi protocol live birth rates per transfer cycle were as high as 40% in patients under 38 years old. The Bondi protocol was more effective as NK activity increased from ‘normal’ to ‘borderline’ to ‘high’. For high NK cell activity levels, live birth rates were over four times higher for women on the Bondi protocol (28%) than those on normal protocols (6%, *P* = .0007).

**Conclusion:**

This study describes a simple and relatively safe immune therapy protocol that may improve IVF success rates in women with evidence of immune dysfunction.

## 1 INTRODUCTION

Repeated IVF failure (RIF) is commonly described as the failure of three consecutive good‐quality embryo transfer cycles,^1^ affecting about 10% of couples undergoing IVF treatment.^2^ Given the enormous financial, physical and psychological burden of IVF, further investigation is warranted and that is the reason why immune testing and therapy has such a long and persistent presence in reproductive medicine.^3^ Women themselves are particularly aware that it is their bodies that have to carry the pregnancy, and dismissing their concerns leads to IVF treatment dropouts,^4^ and engagement with alternative practitioners.^5^ Even if, as some might think, embryo quality is almost entirely responsible for IVF outcomes, unexplained RIF is a very real and distressing condition that requires sensitive and careful investigation. Such an approach guiding empirical therapy may, at the very least, prevent patient ‘drop out’ and so enhance cumulative pregnancy rates.

There is little doubt that the maternal immune system is essential for the establishment and maintenance of pregnancy.^6^, ^7^, ^8^, ^9^ Modification in immune function is necessary to enable implantation of trophoblast cells that are seen as ‘foreign’, and attempts to improve IVF outcomes with adjuvant immune suppressive therapies were first described in 1986.^10^ But immune suppression for everyone was soon shown to be not effective in randomised trials,^11^, ^12^, ^13^ and the maternal immune response was found to be more complex with some activated elements.^6^ Indeed, it has been argued that immune suppression may be detrimental for pregnancy.^8^

Hence, attention has shifted to a more targeted approach to immune therapy.^3^ While the immune system is undeniably a complex web of interacting cells, proteins and hormones, in pregnancy, there does appear to be a particular prominence given to natural killer (NK) cells, which are by far the most common immune cell type in the uterus.^14^ It is still not known whether they assist or inhibit trophoblast invasion, possibly because they do both. The fundamental interaction of polymorphic NK cell killer immunoglobulin receptors (KIR) with the major histocompatibility complex (MHC) tissue recognition antigen HLA‐C on trophoblast cells creates a dynamic limitation on the size of babies.^15^ In evolutionary terms, it is believed that polymorphic interaction enabled our human ancestors to walk upright in spite of a precariously narrow birth canal.^16^

Ninety percent of uNK cells are regulatory CD56+ bright cells secreting cytokines and angiogenic factors that play an active role in trophoblast invasion and remodelling of spiral arteries.^17^ The remaining 10% are potentially cytotoxic CD56+dim cells. This ratio is often cited as a reason for considering uNK cells as, overall, not harmful for pregnancy.^8^, ^18^ But in truth, it is actually not known how the subtypes play out at a local level and what small changes in the ratio could impact on the success of implantation. Mature uNK cells are derived partly from precursors already in the uterus, and partly from trafficking of blood cells.^19^, ^20^ In peripheral blood the ratio of CD56+bright and CD56+dim NK cells is reversed,^21^ and the majority of NK cells that traffic from the blood to the uterus every month is likely to be the CD56+dim subtype.

Peripheral blood (b)NK cell testing would be an appealing assessment tool due to its convenience and non‐invasiveness.^3^, ^16^ Arguments concerning the value of this testing have centred on the link between blood and uterine NK cells.^3^, ^18^ In truth, though it makes no sense to compare numbers of different cell types in different tissues analysed by different laboratory technologies (immunohistochemistry and flow cytometry). But when considering each test independently, it has been shown that ‘high’ levels of activated bNK cells do predict ‘high’ uNK levels.^22^ Moreover, regardless of the relationship with uNK cells, bNK cell assessment may still be a useful marker for immune dysfunction. Pragmatically, high bNK cell activity is associated with RIF,^23^ recurrent miscarriage,^24^ longer periods of infertility, more IVF transfers, more miscarriages and fewer live births.^25^ High bNK activity may identify a female phenotype associated with less cancer,^26^ and greater incidence of endometriosis and thyroid dysfunction.^25^

Using the NK blood test as previously described,^23^, ^24^ women with RIF in this study were treated with an empirical immune protocol of prednisolone and enoxaparin (specifically the low molecular weight heparin brand ‘clexane’, which is widely available in Australia). Following a case report in 2012,^27^ it became known as the ‘Bondi protocol’.^28^ The distinguishing aspects of the protocol are that (1) treatment commences at the beginning of the transfer cycle, (2) prednisolone doses increase to 20 mg daily, (3) clexane is given at a low dose of 20 mg, and (4) therapy continues until 12–16 weeks gestation.

The ‘Bondi protocol’ is a simple, cheap, safe and widely available protocol for immune suppression. Clexane has been commonly used in the past for RIF, vaguely to ‘improve uterine blood flow’ and safety (no known teratogenic effects), although numerous studies have not shown benefit when given on its own and without thrombophilia.^29^, ^30^ The low dose of clexane used in the Bondi protocol deliberately aims for its mild immune suppressive and other potential pro‐implantation effects.^31^, ^32^

Prednisolone is a broad‐spectrum immune suppressant that can reduce uNK cell levels^33^ and suppress bNK cell cytotoxicity.^34^ More importantly, prednisolone has the longest and most extensive published record of immune therapy in IVF, and it is still uncertain whether there is proven benefit from its use or not.^2^ Current consensus is based on trials spread over 30 years during which IVF protocols and patients have changed considerably. In one large metanalysis,^35^ the conclusion was largely based on a single trial, which provided nearly one‐third of all patients, where corticosteroids were administered late in the cycle, and which was published in abstract form only.^36^ Removing that study from the meta‐analysis, nine of the remaining 12 studies indicated favourable outcomes with prednisolone.

When this clinical study was first conceived after the creation of the NK test,^24^ it was common practice to give clexane as an empirical therapy in women with repeated IVF failure. Given the uncertain benefits of prednisolone, and the empirical nature of both therapies, it was decided to give both together. The combination of prednisolone and clexane has subsequently been reported by others, and with beneficial outcomes. A quasi‐randomised controlled trial involving 334 women with unexplained failed ICSI demonstrated significantly improved implantation rates (23.9% vs. 14.7%).^37^ And a small observational trial in 115 women with RIF demonstrated higher live birth rates (26.3% vs. 15.5%) although the difference was not statistically significant.^38^ Neither study assessed NK cell activity.

The aim of this study was to define the success rate of the Bondi protocol in a large cohort assessed with bNK cell testing. It was hypothesised that patients with the Bondi protocol would have had poorer reproductive histories and evidence of immune dysfunction, and would have responded to the therapy to achieve similar success rates as ‘normal’ patients not given the trial therapy. This is the null hypothesis that, at the very least, IVF outcome was not harmed by taking the Bondi protocol.

## 2 MATERIALS AND METHODS

This was a retrospective review of the Bondi protocol in women undergoing embryo transfers by a single clinician (GS) at IVFAustralia between February 2016 and April 2020. This included 1806 transfer cycles across 853 patients: 467 cycles with Bondi protocol treatment and 1339 ‘normal’ cycles without Bondi protocol treatment.

In this clinical study, repeated IVF failure was defined as two or more blastocyst transfer failures. Couples were investigated with a comprehensive endocrine, thrombophilia and autoantibody screen for the women (full blood count, cardiolipin antibodies, lupus anticoagulant, activated protein C resistance, protein C, protein S, antithrombin III, prothrombin gene mutation, Factor V Leiden mutation, MTHFR mutation, thyroid antibodies and hormone, fasting glucose, insulin and homocysteine), karyotype screening for both partners, hysteroscopy and endometrial sampling, and sperm DNA fragmentation testing. After three or more transfer failures, women were offered a further blood test for natural killer activity as described below.

As described elsewhere, fresh IVF/ICSI cycles were routine antagonist (80%), long protocol (6%) or flare protocol (14%). Frozen embryo transfer cycles were routine natural (30%), stimulated (64%) or hormone replacement (6%). There were 11 cycles associated with egg thaws.

Ages reported in this study refers to age of the egg at fertilisation. For fresh cycles, this was the age of the woman at the start of her IVF cycle. For frozen cycles, this was the age of the woman at egg collection and freezing.

For embryo quality analysis, using the Gardner criteria, two categories of ‘good embryos’ and ‘poor embryos’ were formed, combining stage and grade. ‘Good embryos’ were defined as stage 3 and above with AA, AB, BA or BB combinations, or frozen collapsed embryos with higher than 60% cell survival rate. Everything else was defined as ‘poor’.

Clinical notes were used to determine previous transfer history prior to the study timeframe and often at previous clinics. This information was used as background only. Cycle analysis was performed on all cycles done at IVFAustralia in the study period without exception. Many patients continued with therapy after the endpoint of this study.

As previously described,^24^ bNK cell test results consisted of a combination of the total NK cell count as a percentage of lymphocytes and the concentration of activated NK cells. Considering both numbers, there were three clinical categories for NK cell activity: normal, borderline and high. A ‘normal’ NK result was where the total percentage was less than 12% and the activated count was less than 12×10^6^/L. A ‘high’ result was where the total percentage was over 18% and the activated count was over 12×10^6^/L. And a ‘borderline’ result was where one or other marker was not ‘normal’, but the combination did not reach criteria for ‘high’.

Women with a clinical history of repeated transfer failure (at least two as described above) *and* ‘high’ or ‘borderline’ blood NK cell activity were offered empirical therapy with the ‘Bondi protocol’ for their next treatment cycles. In the Bondi protocol, women take 10 mg of prednisolone daily from the start of the cycle. This is increased to 20 mg the day after egg collection or ovulation, and 20 mg clexane injections are started. Prednisolone is continued until 12 weeks of pregnancy or a negative pregnancy test, and clexane injections are continued until 16 weeks of pregnancy or a negative pregnancy test.

### 2.1 Statistical analyses

Statistical analysis was performed on GraphPad Prism software, using Fisher's exact and Mann‐Whitney *U*‐tests.

### 2.2 Ethical approval

The study was approved by the IVFAustralia R&D Committee.

## 3 RESULTS

### 3.1 Cycles

Thirteen patients involving 20 cycles were excluded from analysis due to incomplete information, leaving 1786 cycles over 840 patients as the basis for the study. An overall summary is shown in Table 1. Amongst all the women in the study, the mean age was 37.0, 40% of transfers were fresh, 76% were single embryo transfers and the remainder were all double, 20% of cycles were with euploid embryos, and live birth rate was 27% per cycle.

**TABLE 1:** Overview of database

	All	Bondi	Normal	Intralipid
Cycles	1786	460	1274	52
Mean age^f^ (SD, range)	37.0 (4.4, 24.5‐49.5)	37.3^1^, ^2^ (4.5, 24.5‐49.2)	36.8^1^, ^3^ (4.4, 24.5‐49.5)	39.3^2^, ^3^ (3.6, 28.4‐44.3)
Fresh | frozen (%)	40% | 60%	37% | 63%	41% | 59%	37% | 63%
ICSI | IVF^g^ (%)	62% | 38%	65.5% | 24.5%	61% | 39%	79% | 21%
No. of embryos transferred	1.24	1.34^d^	1.19^4^, ^5^	1.42^e^
PGD tested (%)	20%	22%	19%	27%
Pregnancy per cycle (%)	33%	32%	34%	23%
Live birth per cycle (%)	27%	26%	28%	15%
Miscarriage rate^h^(%)	20%	20%	19%	33%
Multiple pregnancy rate (%)	1.7%	1.7%	1.7%	0%

^a^ Bondi/normal age *P* = .0336.

^b^ Bondi/intralipid age *P* = .0008.

^c^ normal/intralipid age *P* ≤ .0001.

^d^ Bondi/normal transfers *P* ≤ .0001.

^e^ Normal/intralipid transfers *P* = .0003.

^f^Age refers to the age of the egg at fertilisation.

^g^IVF/ICSI are for fresh transfers only.

^h^Calculated based on different between pregnancy and live birth, including both biochemical and clinical pregnancy losses.

There were no significant differences in pregnancy rates or live birth rates between ‘Bondi’ (32%, 26%) and ‘normal’ (34%, 28%) cycles. However, women (or the ages of the eggs at fertilisation) undergoing ‘Bondi’ cycles were significantly older than those undergoing ‘normal’ cycles. ‘Bondi’ cycles also had significantly more double embryo transfers, although the multiple pregnancy rate was low (1.7%) and the same across both groups. In a sub‐analysis of single euploid embryo transfer cycles, the pregnancy and livebirth rates for ‘Bondi’ (41%, 36%, n = 90) and ‘normal’ cycles (43%, 39%, n = 241) were also not significantly different.

An alternative immune therapy – intralipid – was trialled on its own in 52 cycles in this study, and these were excluded from the main analysis of the study. Intralipid cycles were associated with significantly higher mean age and more double embryo transfers. The live birth rate for those intralipid cycles was relatively low (15%) but not significantly different to Bondi cycles (26%). A further 72 cycles had joint therapy of Bondi protocol and intralipid and were regarded as simply Bondi cycles.

Table 2 shows the live birth rates in fresh IVF, fresh ICSI and frozen transfer cycles. The highest success rates were in the frozen groups for both ‘Bondi’ and ‘normal’, and those included all euploid embryo transfers. In a sub‐analysis of women under age 40, there was no difference in IVF, ICSI and frozen success rates in ‘normal’ cycles. But in ‘Bondi’ cycles the ICSI group had a significantly lower live birth rate (19%) than both IVF (50%) and frozen (34%).

**TABLE 2:** Live birth rates per cycle

	Live birth rates	IVF (%)	ICSI (%)	Frozen (%)
Bondi	All (mean age, no. of cycles)	24% (39.2, n = 58)	15% (39.2, n = 110)	30% (36.3, n = 292)
	<40 yrs (mean age, no. of cycles)	50%^a^ (35.5, n = 28)	19%^1^, ^2^ (36.4, n = 64)	34%^b^ (34.8, n = 233)
Normal	All (mean age, no. of cycles)	23%^c^ (38.0, n = 207)	23%^d^ (38.2, n = 319)	31%^3^, ^4^ (35.9, n = 748)
	<40 yrs (mean age, no. of cycles)	32% (35.2, n = 129)	34% (35.1, n = 186)	33% (34.8, n = 629)

^a^ Bondi < 40 yrs IVF/ICSI, *P* = .0048.

^b^ Bondi < 40 yrs ICSI/frozen, *P* = .0215.

^c^ Normal all IVF/frozen *P* = .0199.

^d^ Normal all ICSI/frozen *P* = .0077.

**FIGURE 1:**
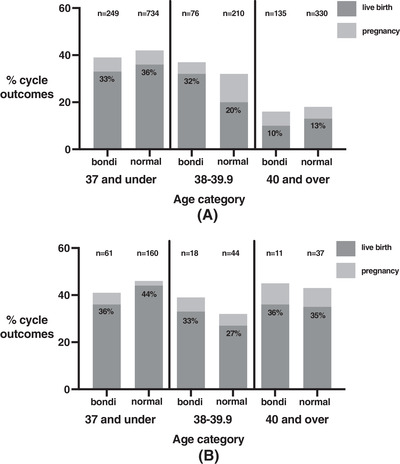
(A) All cycles: positive pregnancy and live birth. (B) PGD single transfer cycles only: positive pregnancy and live birth

Egg age was further examined across three groups: < 38, 38–39.9 and > 40 years. Pregnancy and livebirth rates within each age group are shown in Figure 1A. In both ‘Bondi’ and ‘normal’ groups, livebirth rates were significantly higher in the < 38 group, at 33% and 36%, dropping to 10% and 13% in the > 40 group. Livebirth rates were higher in ‘Bondi’ cycles (32%) for women aged 38–39.9 compared with normal cycles (20%), but this did not reach significance. In euploid transfer cycles, the pregnancy and livebirth rates remained steady across all age groups (Figure 1B). Miscarriages occurred in both ‘Bondi’ cycles and euploid transfer cycles at a rate of 5–9%, with no significant differences.

### 3.2 Patient groups

Excluding nine patients who had intralipid cycles, there were 831 patients (1734 cycles) who were divided into three groups: the ‘Bondi’ group (n = 160) consisting of patients who only ever had ‘Bondi protocol’ cycles, the ‘normal’ group (n = 573) who only ever had ‘normal’ cycles, and the mixed group (n = 98) who had both kinds of cycles. Note this only accounted for cycles completed within the timeframe of this study. Their characteristics are displayed in Table 3.

**TABLE 3:** Outcomes and number of cycles for the three patient groups

	Bondi	Normal	Mixed
Patients	160	573	98
Age	37.1	36.6^a^	37.7^a^
Previous cycles	3.5^2^, ^3^	1.6^b^	2.3^c^
Pregnancy	59% (95)	61% (348)	54% (53)
Live birth	52% (83)	54% (309)^d^	42% (41)^d^
Mean number of cycles in this study	1.76^e^	1.88^f^	3.85^5^, ^6^

^a^ Normal/mixed age *P* ≤ .0001.

^b^ Bondi/normal previous cycles *P* ≤ .0001.

^c^ Bondi/mixed previous cycles *P* ≤ .0001.

^d^ Normal/mixed live birth *P* = .0289.

^e^ Bondi/mixed mean number of cycles *P* ≤ .0001.

^f^ Normal/mixed mean number of cycles *P* ≤ .0001.

During the study timeframe, livebirth rates were 52% for Bondi patients, 54% for normal patients and 42% for mixed patients. Bondi patients had significantly more previous cycles (3.5) than the normal group (1.6) and the mixed group (2.3). During the study timeframe, the mixed group had significantly more cycles (3.85) than both the Bondi group (1.76) and the normal group (1.88).

Combining previous and current therapy, women in the Bondi group had significantly more cycles (5.3) than the normal group (3.5, *P* ≤ .0001). The mixed group had the highest overall number of cycles (6.1).

**FIGURE 2:**
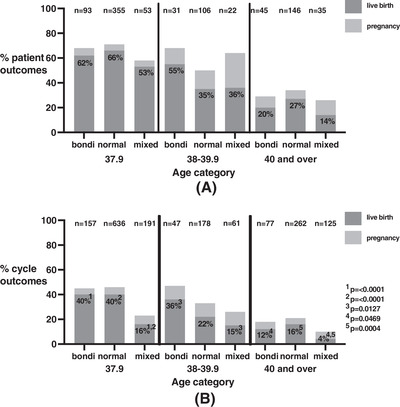
(A) Patient group: positive pregnancy and live birth outcomes per patient. (B) Cycle outcomes according to age and patient treatment groups

The pregnancy and livebirth rates were assessed for the three patient groups in Figure 2A, categorised by age. During the study timeframe, 62% of women on the Bondi protocol in the < 38 group had a live birth, dropping to 55% for ages 38–39.9 and 20% for > 40. This was achieved with cycle success rates of 40% for < 38, 36% for 38–39.9 and 12% for > 40(Figure 2B). This was not significantly different to women on normal protocols.

### 3.3 Live birth outcomes

Overall, 54% (n = 428) of patients achieved a live birth within the timeframe of the study and 46% (n = 369) did not (Table 4). Across all three patient groups (Bondi, normal and mixed), patients who failed had a much higher mean age of 38.7, compared to 35.4 in the live birth group. It was noteworthy that in the Bondi group, patients who failed also had significantly fewer cycles (1.6 vs. 1.9).

**TABLE 4:** Comparing successful and unsuccessful patients

	Bondi protocol only		Normal protocol only		Mixed – Bondi and normal protocols	
	Live birth (n = 83)	No live birth (n = 77)	Live birth (n = 306)	No live birth (n = 249)	Live birth (n = 39)	No live birth (n = 43)
Mean age (SD, range)	35.5^a^ (4.0, 25.6‐44.9)	39.2^a^ (3.7, 28.3‐46)	35.1^b^ (3.8, 24.5‐43.9)	38.3^b^ (4.4, 27.2‐46.7)	35.7^c^ (3.3, 27.6‐44.1)	39.7^c^ (4.8, 24.5‐49.5)
Mean number of cycles (SD, range)	1.9^d^ (1.1, 1‐6)	1.6^d^ (1.1, 1‐6)	1.9 (1.3, 1‐7)	1.8 (1.2, 1‐8)	4.1 (2.1, 2‐9)	4.0 (1.8, 2‐8)

^a^ Bondi age *P* ≤ .0001.

^b^ normal age *P* ≤ .0001.

^c^ mixed age *P* ≤ .0001.

^d^ Bondi cycles *P* = .0246.

### 3.4 Mixed group

Mixed group patients had tried both ‘Bondi’ and ‘normal’ therapies during the timeframe of the study. For this subgroup, embryo quality was assessed as an additional variable. There was no significant difference in the percentage of ‘good’ embryos in Bondi cycles (77.5%) versus normal cycles (78.3%). For all embryos that succeeded, 41/43(95%) were ‘good embryos’; whereas, for embryos that failed, only 206/274(75%) were ‘good embryos’ (*P* = .0015). For Bondi cycles that succeeded, 26/28 embryos (93%) were categorised as ‘good embryos’, compared with 15/15(100%) ‘good embryos’ in ‘normal’ cycles that succeeded.

In this mixed group, 23/38(82%) patients had between one and four failed normal cycles, followed by success with the ‘Bondi protocol’. The other 15 women experienced a mixed approach of ‘Bondi’ and ‘normal’ cycles, with three more patients succeeding on a Bondi cycle, and the remaining 12 succeeding on normal cycles.

### 3.5 NK cell test results

Overall, 554 women in the database had NK cell testing (66%). Clinical interpretation was 46% normal, 39% borderline and 15% high according to reference ranges previously published.^24^

All 98 patients in the mixed group received an NK cell test, 158 patients in the Bondi group received an NK cell test and 298/573(52%) patients in the normal group had an NK cell test. Tables 5, 6, 7, 8 represent patients with NK cell tests only.

**TABLE 5:** Clinical interpretation of NK cell test results

	NK cell test result		
Patient group	Normal NK (n = 253)	Borderline NK (n = 215)	High NK (n = 86)
Normal (n = 298)	68% (204)	29% (85)	3% (9)
Mixed (n = 98)	30% (29)	49% (48)	21% (21)
Bondi (n = 158)	13% (20)	52% (82)	35% (56)

Most patients in the normal group had normal NK cell tests with only 3% having high NK levels (Table 5). In contrast, in the Bondi group, 35% had high bNK cell activity and only 13% had normal bNK cell activity. The mixed group had a more even spread.

Pregnancy outcomes according to NK result and treatment group are shown in Table 6. In the mixed group, livebirth outcomes were even across all three NK groups (38‐44%). In the ‘Bondi’ group, the livebirth rates increased from normal NK (40%) to borderline NK (58%) and high NK (49%), though this was insignificant. In the ‘normal’ group, the livebirth rates decreased from normal NK (50%) to borderline NK (42%) to high NK (11%). The livebirth rate of 11% in the high NK group was significantly lower than the 50% in the normal NK group (*P* = .036), though sample size was small.

**TABLE 6:** Outcomes associated with NK cell results per patient group

		Normal NK	Borderline NK	High NK
Bondi group (n = 158, 278 cycles)	Pregnancy	50% (10/20)	61% (50/82)	60% (34/56)
	Live birth	40% (8/20)	58% (47/82)	49% (28/56)
	Mean number of cycles (SD, range)	1.8^b^ (1.4, 1‐6)	1.7^b^ (1.0, 1‐5)	1.8 (1.2, 1‐6)
Normal group (n = 298, 604 cycles)	Pregnancy	57% (117/204)	49% (42/85)	33% (3/9)
	Live birth	50%^a^ (103/204)	42% (36/85)	11%^a^ (1/9)
	Mean number of cycles (SD, range)	2.2 (1.4, 1‐8)	1.8 (1.4, 1‐8)	1.3 (0.7, 1‐3)
Mixed group (n = 98, 377 cycles)	Pregnancy	62% (18/29)	48% (23/48)	57% (12/21)
	Live birth	38% (11/29)	44% (21/48)	43% (9/21)
	Mean number of cycles (SD, range)	3.8 (1.7, 1‐8)	4.0 (2.1, 1‐9)	3.5 (2.0, 1‐9)

^a^ Live birth normal/high *P* = .0356.

^b^ cycles normal/borderline *P* = .0095.

In women with high NK cell levels, livebirth rates in the ‘Bondi’ group were higher (49%) than the normal group (11%), but this did not account for the higher number of cycles in the Bondi group (1.8 vs. 1.3).

Cycle data and NK test results were further explored in Table 7. Within the mixed group, there was a trend favouring the ‘Bondi’ protocol in the borderline and high NK groups. Livebirth rates were 12% and 8% for ‘Bondi’ and ‘normal’ cycles respectively for women with normal bNK activity, 17% and 8% for those with borderline bNK activity, and 23% and 5% for those with high bNK activity. This was statistically significant.

**TABLE 7:** Outcomes associated with NK cell results per cycle

Mixed group – Bondi cycles vs. normal cycles						
	Normal NK		Borderline NK		High NK	
	Pregnancy	Live birth	Pregnancy	Live birth	Pregnancy	Live birth
Bondi cycles	20% (10/51)	12% (6/51)	25% (23/93)	17% (16/93)	26% (9/35)	23%^a^ (8/35)
Normal cycles	18% (11/60)	8% (5/60)	14% (14/100)	8% (8/100)	13% (5/38)	5%^a^ (2/38)

Bondi group vs. normal group						
	Normal NK		Borderline NK		High NK	
	Pregnancy	Live birth	Pregnancy	Live birth	Pregnancy	Live birth
Bondi	28% (10/36)	22% (8/36)	39% (56/143)	36% (51/143)	40% (40/99)	30% (30/99)
Normal	32% (142/440)	26% (114/440)	29% (44/152)	25% (38/152)	25% (3/12)	8% (1/12)

^a^ Mixed group Bondi/normal high NK live birth rate *P* = .0412.

A similar trend was observed comparing the ‘Bondi’ and ‘normal’ groups. In the normal NK group, livebirth rates were 22% for the Bondi group and 26% for the normal group. This was reversed in the borderline NK group, with livebirth rates of 36% and 25% for Bondi and normal cycles, and even greater divergence in the high bNK group with rates of 30% and 8%.

In a sub‐analysis of women under 40 years of age, this trend of increasing success with the Bondi protocol with increasing NK cell activity was even stronger. The difference in success rates between ‘Bondi’ and ‘normal’ cycles increased from ‐2% (29% vs. 31%) for normal NK, to 13% (44% vs. 31%) for borderline NK, to 26% (37% vs. 11%) for high NK. This was in favour of Bondi protocol therapy and amounted to over a three‐fold difference.

**FIGURE 3:**
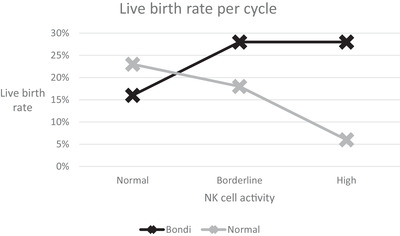
Live birth rate per cycle trend

Combining cycles from all three patient groups, larger numbers were obtained. Outcomes associated with all ‘Bondi’ and ‘normal’ cycles are shown in Table 8. In the normal NK group, livebirth outcomes were better in ‘normal’ cycles (23%) than ‘Bondi’ cycles (16%), though not significant. However, there was a striking and highly significant switch in women with abnormal bNK activity. In those with borderline bNK results, the livebirth rate was significantly higher with the Bondi protocol (28%) than normal protocols (18%, *P* < .01). And in those with high bNK results, the livebirth rate with the Bondi protocol was also 28%, but in normal protocols it was only 6% (*P* < .001). In this analysis, 672 cycles were analysed in women with borderline and high bNK results, 184 of them in the high group. The impact of the Bondi protocol in women with increasing bNK activity, culminating in an over four‐fold difference in livebirth outcomes for the high NK group is illustrated in Figure 3.

**TABLE 8:** Outcomes associated with Bondi cycles and normal cycles

	Normal NK		Borderline NK		High NK	
	Pregnancy	Live birth	Pregnancy	Live birth	Pregnancy	Live birth
Bondi cycles	23% (20/87)	16% (14/87)	33%^a^ (79/236)	28%^b^ (67/236)	37%^c^ (49/134)	28%^d^ (38/134)
Normal cycles	30% (153/500)	23% (119/500)	23%^a^ (58/252)	18%^b^ (46/252)	16%^c^ (8/50)	6%^d^ (3/50)

^a^ borderline NK pregnancy rate *P* = .0117.

^b^ borderline NK live birth rate *P* = .0098.

^c^ high NK pregnancy rate *P* = .0072.

^d^ high NK live birth rate *P* = .0007.

## 4 DISCUSSION

This retrospective review of live birth outcomes is the largest study yet published of prednisolone and clexane therapy for women with repeated IVF failure. This therapy was first described in 2012 and has been called the ‘Bondi protocol’.^27^, ^28^ Two hundred and fifty‐eight women were given the Bondi protocol empirically based on a history of RIF and bNK cell test results. They were compared with 573 women who did not have the Bondi protocol. The study included fresh IVF and ICSI cycles, and frozen transfer cycles with euploid and untested embryos. The critical factor of egg age was accounted for by using the age of the egg at fertilisation. Previously, the largest study comprised 334 patients in total, with 148 treated with prednisolone and clexane over one cycle only.^37^ That study also reported higher pregnancy and implantation rates in women given the immune therapy, but it did not assess livebirth rates, and there was no immune investigation.

The Bondi protocol produced equivalent success rates as normal protocols (26% vs. 28%), with no differences across age groups and euploid embryo transfer cycles. In women under age 38, 62% achieved a live birth with the Bondi protocol during the timeframe of this study. That was achieved with a cycle success rate of 40%. These outcomes compare favourably with average outcomes reported by the national database in Australia and New Zealand (ANZARD), with livebirth rates of 40.4% per cycle for women under 30, and 25.6% for those aged 35–39 years.^39^ These data argue against the hypothesis that immune suppression such as produced by the Bondi protocol may be detrimental for implantation.^8^

On the contrary, the Bondi protocol did appear to compensate for potentially worse expected outcomes in women with RIF who were given it. Bondi patients were older (37.3 vs. 36.3 years), had more previous cycles (3.5 vs. 1.6) and had higher NK cell levels suggestive of immune dysfunction. And even more importantly, the Bondi protocol was increasingly more effective with increasing NK activity. For high NK levels, the cycle success rate on Bondi therapy (28%) was significantly higher than normal therapy (6%), demonstrating over a four‐fold difference in livebirth outcomes. This study has therefore indicated an association between bNK test results and IVF outcome, as well as the benefit of treatment with the Bondi protocol.

‘Bondi’ and ‘normal’ cycles were comparable in all aspects other than mean age and number of embryos transferred. Advancing maternal age or, more precisely, the age of the egg at fertilisation, has the most powerful impact on IVF outcome.^1^ Hence, the equivalence of ‘Bondi’ cycle outcomes is all the more striking as those cycles used significantly older eggs than ‘normal’ cycles.

‘Bondi’ cycles were associated with significantly more double embryo transfers (34% vs. 19%), giving a mean embryo transfer number of 1.34 compared to 1.19 in ‘normal’ cycles. It is unlikely that this was the cause of better IVF outcomes with the Bondi protocol, and more likely reflects poorer embryo quality. This was illustrated in the mixed group where the Bondi protocol cycles actually had slightly worse embryo quality (although not statistically significant), and by the fact that the higher transfer embryo number in ‘Bondi’ cycles did not result in more multiple pregnancies. Similarly in the ANZARD database, the livebirth rate per transfer cycle was 26.4% for single embryo transfers and 16.5% for double embryo transfers,^39^ almost certainly reflecting clinical practice of transferring more embryos in more difficult cases. It is unlikely that there was much gain from double embryo transfers, and instead the success rates are likely from use of the Bondi protocol.

Prednisolone was chosen for the Bondi protocol as it is affordable, widely available and easy to administer. Several studies indicate benefit with prednisolone use for women with recurrent miscarriage,^27^, ^33^, ^40^ and with RIF.^12^, ^28^, ^38^ It is proposed to reduce NK cell activity,^2^ with 20 mg of prednisolone sufficient to reduce both bNK^34^ and uNK^33^ cell numbers and activity.

Low‐dose clexane also has immune suppressive properties,^31^, ^32^ although it has not been shown to be beneficial on its own except in women with thrombophilia.^29^ At the time of the creation of the Bondi protocol, clexane was a commonly used adjuvant that was given on an empirical basis in women with RIF. It is currently not known whether it actually contributes to the overall impact of the Bondi protocol.

Depending on resources, offering women with RIF empirical immune therapy with the Bondi protocol is certainly an option. In this study, there was no difference in livebirth rates overall, and no significant difference in live birth rates in women with normal NK cell tests. However, it should be noted that success rates in those women were slightly lower than those on ‘normal’ protocols’, and with larger numbers that difference may be significant. In other words, in the absence of an immune test, or in the context of a normal immune test, the Bondi protocol should not be offered, or given with caution. In that situation, the potential harm from reduced success rates and teratogenic effects may outweigh any imagined benefit.

Immune investigation in women with RIF is still considered controversial by many.^8^, ^18^ The reasons are related to the paucity of good data, the excessive interpretation of technically poor tests, and the bewildering array of possible immune therapies, which have never been directly compared and have never been convincingly shown to be beneficial.^3^ Hence, the need for caution. But that does not mean dismissing the concept entirely as many women are likely to gain from the approach.

It is important to establish each test and therapy in its own right. It is highly unlikely that intralipid, for example, has the same effect on a woman's immune system as the Bondi protocol. In this study, a small comparison was possible between the Bondi protocol and intralipid. Although women given intralipid were significantly older, it is not obvious that intralipid would be a comparable alternative to the Bondi protocol. However, the intralipid sample was small (n = 52) and other studies have certainly been more encouraging for further exploration as an alternative.^41^, ^42^

Immune testing should continue to be explored as a means of determining the most likely patients to gain from immune therapy. The need for testing has other important and often underappreciated benefits. There is considerable evidence that women are simply not satisfied by the basics of IVF.^43^ In addition to cycle success rates, they consider costs, time invested, and support given in various ways by clinics, websites and hands‐on practitioners. They seek out alternative therapies^5^ and immune testing of some kind or other is widely requested even in the absence of evidence.^44^ Perhaps women feel disempowered by the IVF process and they want to play a more active part in their future pregnancy. Or perhaps they have a stronger belief in the importance of the environment that an embryo is transferred into than many of their clinicians. But on a purely practical level, the ‘numbers game’ approach of IVF is simply too difficult for many. It is expensive and emotionally exhausting. And thus, it could be argued that immune testing needs to be an active research programme in every IVF clinic.

The blood NK test used in this study was created with normal controls to provide a realistic approach for defining women as being ‘normal’ or not.^24^ Previous studies used relatively low reference ranges,^45^ and few have used additional functional tests such as the CD69 activation marker used in this study.^46^, ^47^ The combination of ‘percentage of lymphocytes’ and ‘activation’ provides a more robust NK diagnosis, but the test requires specific flow cytometry expertise and is not widely available. The test has previously demonstrated that women with RIF have significantly higher bNK activity than normal controls,^23^ consistent with others.^46^, ^47^ As the test was provided to larger numbers of women, we also demonstrated that women with borderline and high levels had increasing numbers of transfer failures, more miscarriages and fewer live births.^25^

It is clear that the uterus is the critical place that determines success or failure of embryo implantation, and it is entirely appropriate for clinicians to focus on uterine tests as more ‘useful’. However, aside from the invasiveness of endometrial sampling, the available technologies for assessing immune function in the endometrium are currently simply inadequate or still unproven in the clinical setting.^16^ Blood NK testing, on the other hand, was first described in 1995 and continues to have widespread appeal.^3^ In this study, women with high bNK cell activity who did not have any immune therapy had a fourfold lower cycle livebirth rate than those with normal NK cells.

The Bondi protocol increased live birth rate in women with borderline NK cell activity (28% vs. 18%) and even more strikingly in women with high NK cell activity (28% vs. 6%). The success of the Bondi protocol was achieved with a mean of 1.9 cycles, comparable to those who succeeded with normal protocols. In the mixed patient group, 23 women succeeded on their first cycle with the Bondi protocol after up to four ‘normal’ protocol cycles. An interesting finding in the Bondi group (women only treated with the Bondi protocol) was that those who failed to achieve a live birth during the timeframe of the study had significantly fewer attempts than those who succeeded. It is well recognised that success rates for both patients and clinics are severely impacted by women dropping out of treatment.^4^ This study provides further evidence that simply encouraging women to continue is one of the most significant contributions a clinician can make.

In this study the miscarriage rate (including biochemical losses) was not significantly different between ‘Bondi’ and ‘normal’ protocol cycles (about 20% overall). In fact much of the evidence for potential benefit of prednisolone has come from studies of women with recurrent miscarriage.^33^ In one such randomised double‐blind placebo controlled trial involving 180 women, prednisolone produced a 61.1% decrease in miscarriage risk.^40^ It is possible that the live birth benefit of the Bondi protocol was primarily through an effect of reducing miscarriage, although this did not seem to be the case as pregnancy rates mirrored live birth rates exactly. Thus, it is hypothesised that the impact of prednisolone on the development of the endometrium throughout the cycle produced changes that facilitated attachment as well as early implantation of the embryo. Prednisolone certainly has multiple effects on many cell types in the endometrium, and we need not assume that its benefit is directed solely on uterine NK cell numbers.^48^ The hypothesis that prednisolone affects endometrial‐embryo signalling needs to be explored.

This study confirms and may provide some explanation for previous reports that prednisolone was beneficial in women undergoing IVF but not ICSI.^35^, ^49^ In women under 40, livebirth rates with the Bondi protocol were 50% for IVF and only 19% for ICSI. An explanation for this might be that the ICSI group would have been dominated by male factor issues, while those with solely immune dysfunction would have been more likely to be in the IVF group. Consistent with that, women undergoing IVF did have higher mean NK levels (14.4%) than those undergoing ICSI (13%, *P* < .05). Hence, the IVF group as a whole would be more likely to gain from immune therapy.

Aside from effectiveness, there are many reasons for clinicians to continue to be cautious with the Bondi protocol. Some women dislike side effects such as reduced sleep, palpitations and fluid retention. With longer‐term use, there are the more serious risks of diabetes and osteoporosis. Hence, the Bondi protocol is given specifically for a cycle and first trimester, and it is essential that patients are not prescribed it on a medium‐term basis, ‘while they are going through cycles of IVF’. The prednisolone dose must be reduced whenever possible, with rest periods between failed cycles. On the foetal side, numerous studies on potential teratogenicity of prednisolone have been largely reassuring,^50^, ^51^, ^52^ but there is a possible association with isolated foetal oro‐facial cleft with an odds ratio risk of 3.4.^8^, ^53^ This represents an absolute increased risk from 0.1% to 0.3‐0.4%. In another study of 262 pregnancies exposed to glucocorticoids in the first trimester, there was an increase in major anomalies from 2.6% to 4.6%, but the increase was not statistically significant and there were no cases of cleft palate.^54^ In our study of 120 livebirths with the Bondi protocol, there were no foetal abnormalities reported, but a much larger study would be required to detect the size of risk previously described. The possible small teratogenic risk is certainly a reason for caution, and is only acceptable with demonstrable benefit provided by this study and future trials.

The final argument against immune therapy is that it may cause harm in the form of false hope, and associated financial and emotional stress.^18^ Hope is a critical factor for all IVF patients, and immune testing gives many a reason to continue. This in itself can be the difference between having a baby and remaining childless. The Bondi protocol is well tolerated and does not take away chance in the form of success rate. But even more, it significantly improves success rates in women with immune dysfunction.

The main limitation of this study is that it is retrospective and therefore prone to bias. But the effect of the Bondi protocol on women with high blood NK cell activity is so significant that, on current evidence, it should be considered as empirical therapy for RIF. In terms of benefit demonstrated in this paper, it compares favourably with many other potential adjuvant therapies used in IVF. In women with high NK cells, the ‘number needed to treat’ (NNT) with the Bondi protocol was 4.5. In terms of pregnancy outcomes, this compares with an NNT of 5.9 for ovulation induction therapy with letrozole, 6.3 for varicocele repair, 7.8 for antioxidant therapy, 8.4 for surgical treatment of mild or moderate endometriosis^55^ and 9 for acupuncture.^56^ Even the endometrial receptivity assay (ERA), commonly used in RIF, was recently calculated to have an NNT of 7.2 for frozen embryos and 9.5 for fresh embryos.^57^ And while IVF specialists have broadly accepted the advantage of frozen rather than fresh embryo transfers in women overstimulating with polycystic ovary syndrome, the NNT for that management decision is 14.^58^ In other words, the Bondi protocol for high blood NK cell activity may be an easier and more effective approach to many commonly used strategies in management of infertility. And it is also noteworthy that the benefit of treating women with ‘borderline’ NK cells, increasing livebirth rates from 18% to 28%, also gives an NNT of only 10.

Nevertheless, the Bondi protocol still needs more rigorous testing in prospective randomised controlled trials (RCTs). Using the outcomes in this study, an RCT with 90 women (45 in each arm) would demonstrate a significant difference at *P* < .05 and 80% power. In this and previous studies, ‘high’ blood NK cell activity was identified in 15% of women. This would mean that 600 women with RIF would need NK blood screening. In fact such an RCT has already been performed although it has not, as far as we are aware, been published.^59^ Presented as an abstract and oral presentation at ESHRE in Stockholm in 2011, Alhalabi et al. reported that 112 women undergoing ICSI were identified as having high blood NK cell activity by the CD69 marker, and randomised to receiving prednisolone (58) or no immune therapy (54). The clinical pregnancy rate in the prednisolone group was significantly higher (48.3% vs. 29.6%), although livebirth rates were not reported. Another randomised trial of prednisolone in women with RIF has been registered and results are awaited.^60^

Immune therapy in reproductive medicine has a long history of excessive claims to be ‘the answer’ and equally excessive counter claims that it is ‘useless’. The reality is that it is, at best, just one possible factor out of many that influence the outcome of IVF. Most of those factors, such as the true genetic potential of an embryo, are almost always unknown. In this study, the investigation and potential (although unproven) benefit of therapy were the reasons for women who had been crushed by repeated failure to try again. In itself, that is a success of the Bondi protocol, as the hope led to successful attempts and normal births. In any unproven medical intervention, the first goal is to demonstrate non‐inferiority, which this study has very patently done. In retrospective data, the suggestion of a four‐fold improvement in outcomes with the Bondi protocol in women with high bNK activity is striking and needs further investigation. But it is not ‘the answer’, even for those women with high bNK activity. Immune therapy must be applied cautiously, in selected patients, *after* an assessment and appropriate management of every other possible factor in the IVF process. The Bondi protocol should only be considered as part of a much broader holistic and evidence‐based approach to IVF.

Finally, the combination of prednisolone and clexane (the ‘Bondi protocol’ as described in this study) evolved empirically. It is not known whether both drugs are necessary for the impacts described, and further studies would be needed to investigate that. But it is worth noting that, in women with repeated IVF failure, immune therapies used in combination do appear to be more beneficial than single therapies used on their own.^61^

## 5 CONCLUSION

This retrospective study found that the Bondi protocol (prednisolone and clexane) given to women with RIF resulted in an equivalent live birth rate to normal treatment controls without RIF. It is hypothesised that those women on Bondi protocol treatment would have performed significantly worse without the Bondi protocol. Further, high blood NK cell activity may be an immune marker for lower livebirth outcomes, and the Bondi protocol appears to be a highly effective targeted therapy for those women. This study provides a strong basis for a prospective randomised controlled trial. Ongoing observations and future trials are also necessary to ensure the safety of the Bondi protocol for both mothers and their babies.

## CONFLICT OF INTEREST

The blood NK test is provided through IVFAustralia at a cost of $295.

## Data Availability

The data that support the findings of this study are available from the corresponding author upon reasonable request.
